# Molecular Analysis of Salivary and Lacrimal Adenoid Cystic Carcinoma

**DOI:** 10.3390/cancers16162868

**Published:** 2024-08-17

**Authors:** Sarah Powell, Karina Kulakova, Katie Hanratty, Rizwana Khan, Paula Casserly, John Crown, Naomi Walsh, Susan Kennedy

**Affiliations:** 1 Research Foundation, Royal Victoria Eye and Ear Hospital, Adelaide Road, D02 XK51 Dublin, Ireland; 2 National Ophthalmic Pathology Laboratory, D04 T6F6 Dublin, Ireland; karina.kulakova4@mail.dcu.ie (K.K.); susan.kennedy@rveeh.ie (S.K.); 3 School of Biotechnology, Dublin City University, D09 V209 Dublin, Ireland; 4 St. Vincent’s University Hospital, D04 T6F4 Dublin, Ireland

**Keywords:** adenoid cystic carcinoma, *NFIB-MYB*, *NOTCH*, DNA damage repair, epigenetic modifications

## Abstract

**Simple Summary:**

Adenoid cystic carcinoma is a rare but devastating disease. Currently, treatment options are very poor, and ACC does not respond well to conventional chemotherapy or radiation therapy and has a high rate of recurrence or metastasis. The molecular drivers that govern disease pathology are currently poorly understood. We conducted a molecular analysis of adenoid cystic carcinoma of the salivary and lacrimal glands in order to better understand these mechanisms and pave the way for the development of future therapeutics.

**Abstract:**

Adenoid cystic carcinoma (ACC) of head and neck origin is associated with slow but relentless progression and systemic metastasis, resulting in poor long-term survival rates. ACC does not respond to conventional chemotherapy. Determination of molecular drivers may provide a rational basis for personalized therapy. Herein, we investigate the clinical and detailed molecular genomic features of a cohort of patients treated in Ireland and correlate the site of origin, molecular features, and outcomes. Clinical and genomic landscapes of all patients diagnosed with ACC over a twenty-year period (2002–2022) in a single unit in Ireland were examined and analyzed using fluorescence in situ hybridization, DNA sequencing, and bioinformatic analysis. Fourteen patients were included for analysis. Eleven patients had primary salivary gland ACC and three primary lacrimal gland ACC; 76.9% of the analyzed tumors displayed evidence of *NFIB*-*MYB* rearrangement at the 6q23.3 locus; 35% had mutations in *NOTCH* pathway genes; 7% of patients had a *NOTCH1* mutation, 14.3% *NOTCH2* mutation, and 14.3% *NOTCH3* mutation. The presence of epigenetic modifications in ACC patients significantly correlated with worse overall survival. Our study identifies genetic mutations and signaling pathways that drive ACC pathogenesis, representing potential molecular and therapeutic targets.

## 1. Introduction

Adenoid cystic carcinoma (ACC) is a rare secretory gland carcinoma, with an incidence of three to four patients per million worldwide [1,2]. ACC represents only 1% of head and neck cancers [1]. Its main sites of origin are the major and minor salivary glands. ACC in the lacrimal gland is exceptionally rare but still is the most common lacrimal gland malignancy, accounting for 66% of all carcinomas of the lacrimal gland [3,4]. It has a deceptively low-grade histological appearance and is characterized by indolent yet relentless disease progression [5], often recurs and/or metastasizes [6]. Distant metastasis has been documented to occur in over 50% of completely surgically excised ACC tumors, possibly due to occult perineural invasion [7]. Long-term survival rates of ACC are poor and range between 23 and 40% [8]. Clinical symptoms differ depending on tumor location; most frequently, pain, discomfort, and a slow-growing mass are observed. Complete surgical resection is the treatment of choice for primary ACC patients with adjuvant radiotherapy [1]. Due to the rarity of ACC, the molecular drivers of the disease are poorly understood, and no effective systemic therapies have been developed thus far. 

ACC can have three distinct histological growth patterns on hematoxylin and eosin (H&E) staining, namely tubular, cribriform, and solid [9]. ACC is often biphasic, consisting of more than one pattern in variable proportions. Patients are graded based on the degree of solid growth component in their tumor. This grading can significantly predict patient outcomes, with the best prognosis seen in the tubular pattern and the worst in the solid variant [10,11]. ACC is characterized by perineural invasion (PNI) and is commonly observed in the absence of vascular or lymphatic invasion [12]. PNI is a common finding in ACC and can be considered a potential route for tumor cell propagation.

Somatic, non-germline involving aberrations at a cellular level drive ACC pathogenesis [13]. ACC is characterized by an *MYB-NFIB* gene fusion occurring most commonly via the t(6;9)(q23;p23) translocation, reported in 64% of all ACC tumors [1]. This fusion is the unique genomic hallmark of ACC, which results in *MYB* upregulation. It has not been described in other tumor types and is, therefore, a highly sensitive disease biomarker whose overexpression is considered a key oncogenic driver of ACC pathogenesis [2]. The transcriptional activator *MYB* is overexpressed in ACC tumors, leading to pro-tumorigenic cellular processes such as migration, cellular adhesion, cellular proliferation, tumor growth, and angiogenesis. Andreasen et al. reported that *NFIB* is mutated in salivary gland and breast ACC tumor samples and observed that salivary gland, breast, and lacrimal gland ACC were genetically similar [14]. 

*MYB*, an important transcriptional activator and oncogene, is located on chromosome 6q23 [15]. *MYB* overexpression results in several pro-tumorigenic processes, such as cellular growth and differentiation, angiogenesis, and regulation of growth factors [16]. Both *MYB* protein and *MYB* mRNA have been shown to be upregulated in ACC. Conversely, *MYB* is not typically found in non-neoplastic glandular cells [17]. Overexpression of *MYB* can occur through various mechanisms, including selective amplification at the *MYB* locus, and is observed in tumors with and without specific gene fusions [2]. Fluorescence in situ hybridization (FISH) and PCR testing are common methods used to identify chromosomal translocations [18]. In particular, FISH analysis utilizes dual-color ‘break-apart’ probes in order to detect rearrangements in *MYBL1*, *MYB*, and *NFIB* [19]. 

*NOTCH* signaling, DNA damage repair gene mutations, and epigenetic modifications have also been reported in ACC pathogenesis [1,4,20,21,22]. Mutations in the *NOTCH* signaling pathway are found in 11–29% of ACC patients, and it is thought that these mutations may promote ACC pathogenesis by upregulating pro-tumorigenic processes [23]. Aberrant *NOTCH* expression has been reported to be associated with R/M ACC tumors and is associated with a more severe disease phenotype, worse overall survival rates, and poor disease prognosis [24,25]. An attractive therapeutic target, there are several ongoing clinical trials aimed at developing *NOTCH* inhibitors, including brontictuzumab and CB-103, both *NOTCH* 1 inhibitors, which have demonstrated promising disease stabilization data thus far [26,27]. 

Epigenetic modifications are associated with ACC pathogenesis. Chromatin remodeling mutations have been reported in up to 50% of ACC tumors. Mutated chromatin remodeling genes include *ARID1A*, *CREBBP*, an epigenetic modifier gene, and *KDM6A* [28]. *KDM6A*, which plays a role in histone demethylation, has been associated with R/M disease [29]. There is a growing body of evidence that TERT-promoter genes are present in ACC patients without *MYB/MYBL1* fusions or *NOTCH* pathway mutations, indicating alternative tumorigenesis pathways [30]. 

ACC remains both a diagnostic and treatment challenge for head and neck surgeons and oncologists, and the molecular mechanisms that underscore disease pathogenesis are only beginning to emerge. Therefore, there is an unmet, urgent need to develop targeted systemic therapy to treat ACC. Compared to ACC in other anatomical sites, and despite a similar genomic landscape, lacrimal gland ACC has a particularly poor prognosis due to high rates of R/M, perineural invasion, and local infiltration of soft-tissue and bone [31,32,33]. Five-year survival rates of lacrimal gland ACC are <50% and ten-year survival rates have been reported to be as low as 20% [34,35].

The aim of this study is to report on the clinical and genomic landscape of head and neck ACC in our patient cohort and to correlate genetic mutations with clinic–pathologic characteristics and outcomes.

## 2. Methods and Materials

### 2.1. Clinical Data Collection

The archives of the National Ophthalmic Pathology Laboratory were accessioned to identify cases of Head and Neck ACC over a 20-year period (2002–2022). Sixteen patients with a histologically confirmed diagnosis of ACC were identified. Their clinical and histopathological data were reviewed using charts, patient information systems, and the National Death Registry. The clinicopathological features evaluated included patient age, sex, tumor histology pattern, R/M, and overall follow-up survival time. Disease-free survival was determined as either progressed (metastasis or recurrence) or disease-free. Follow-up periods for patients were determined from their date of diagnosis to either their last follow-up appointment or death, as per patient records. Ethical approval for the study was obtained from the Ethics and Medical Research Committee of the Royal Victoria Eye and Ear Hospital, the Committee and Council of the Hospital, and the study was performed under the tenets of Helsinki [36].

### 2.2. Sample Preparation

Tumor samples were histologically confirmed via assessment of their respective formalin-fixed paraffin-embedded (FFPE) histology tissue blocks. Blocks were cut into H&E slides to determine the presence of neoplastic cells in each respective block. These blocks were sectioned using microtomy and stained with H&E [18]. The available matching normal FFPE blocks were also retrieved for analysis. FISH studies were performed on whole tumor sections. For molecular studies, microdissection was carried out via microtomy, and the FFPE scrolls were placed into Eppendorf tubes, which were subsequently subjected to whole-exome sequencing (WES).

### 2.3. FISH Analysis

ZytoLight SPEC *MYB* Dual Color Break Apart Probe (ZytoVision GmbH, Bremerhaven, Germany) was utilized to detect MYB rearrangements. *NFIB* and *MYBL1* rearrangements were observed using Custom-designed SureFISH NFIB and *MYBL1* Break Apart probes. The human genome (hg) build 19 was utilized for the chromosomal locations of the custom *NFIB* break-apart probe oligos, chr9:13740671-14140560 and chr9:14340306-14740560, and the *MYBL1* break-apart probe, chr8:67076230-67474559 and chr8:67526335-68426199. 

### 2.4. DNA Sequencing

WES was performed on the tumors of 16 patients with matched normal available for three patients using the Illumina NovoSeq6000 platform (Illumina, San Diego, CA, USA). DNA extracted from normal and tumor samples were fragmented into 180–280 bp reads via random shearing, which were subsequently subjected to end repair, A-tailing, and ligation with Illumina adapters. DNA was purified and isolated from the tumors and normal specimens using the AllPrep DNA/RNA FFPE Kit (Qiagen, Hilden, Germany). Quality control (QC) was performed with Qubit (Thermo Fisher, Waltham, MA, USA). The samples passed QC. Libraries were generated in Cegat using 50 ng of the sample via Twist Human Core, RefSeq, and Mitochondrial Panel (Twist Bioscience, South San, CA, USA) preparation panel (‘Exome Sequencing’, 2023) [6]. The NovoSeq6000 platform performed pair-ended sequencing, resulting in 100 base pairs at each end of the fragments. Results were available for fourteen patients. 

### 2.5. Bioinformatic Analysis

As a QC step before analysis, the depth and coverage of the sequenced samples were interrogated, thresholds of 100× and 45% were set, and it was decided whether they had sufficient coverage and depth to yield subsequently valid results. All sequenced samples passed this QC step. Another QC process involved assessing the quality of raw reads using quality scores generated by the sequencing platform. 

Raw variant call format (VCF) files were remapped from the hg38 build to hg19 using NCBI’s genome remapping service [37]. The single nucleotide polymorphisms (SNPs) and short insertions and deletions (indels) raw VCF files were combined into a large VCF file per individual patient. Mutation annotation format (MAF) files were created for all samples using the vcf2maf pipeline in the command line. The pipeline annotated VCFs using Ensembl’s integrated variant effect predictor [38]. Tumor and normal matched MAF files were combined with germline mutations removed, leaving only somatic variants. To reduce the number of mutations to be analyzed and remove background sequencing noise, several filters were introduced to the final MAF file. The files were filtered following the criteria demonstrated in Table 1, with blanks retained in each category, resulting in an approximate 10-fold reduction in the tumor mutational burden (TMB). The Maftools package in R version 4.0.2 was used to visualize the genomic landscape [39].

Kaplan–Meier (KM) survival analysis was performed in R to calculate survival [11]. Patients were also stratified by their signature and clinical features to investigate whether there are differences in survival between various subgroups. Time was defined in months for all survival plots. The resulting graphs and statistical tests were analyzed to determine the significance of the outcomes and relationships within the groups. A log-rank test was applied to assess the statistical significance of the findings and results provided by the KM method. 

## 3. Results

Complete follow-up was not available for all patients with confirmed ACC diagnosis. Therefore, overall survival status was described as either dead or alive/censored, Table 2. Of the fourteen, ten are male, and four are female. Clinical data were analyzed for fourteen patients with available WES results. At the time of analysis, eight patients were deceased. The cause of death was ACC for seven patients, and one patient died from other unrelated causes. The mean age was forty-three years, with a median of forty-two years. The tumors originating in salivary glands were found in both the major (4) and minor (8) glands. Three patients had a local recurrence, and five patients developed systemic metastasis during a median follow-up period of twenty-five months. The sites of metastasis included cervical lymph nodes (1), pulmonary (5), renal (2), and splenic (1). The treatment course is known for eleven patients; six received complete surgical resection and adjuvant radiotherapy, three underwent complete surgical resection only, and two received radiotherapy only. Table 3 describes patient demographics of the FISH data analysis

### 3.1. FISH Studies 

FISH analysis was performed to determine the status of *MYB* and *NFIB* genes. Results were available for twelve of fourteen patients; 83.3% (*n* = 10) of the tumors analyzed displayed *MYB* rearrangements at the 6q23.3 locus; 75% (*n* = 9) had *NFIB* rearrangements, and 17% (*n* = 2) of tumor samples did not have rearrangements of *MYB* or *NFIB* genes on FISH analysis. *MYBL1* rearrangements and translocations were not present in any tumor sample analyzed. The two patients negative for *MYB* and *NFIB* translocations were also negative for *MYBL1*. *MYB* rearrangements without *NFIB* occurred in 8.3% of patients. 

#### Whole Exome Sequencing (WES) Single Nucleotide Variant (SNV) Analysis

**Figure 1:**
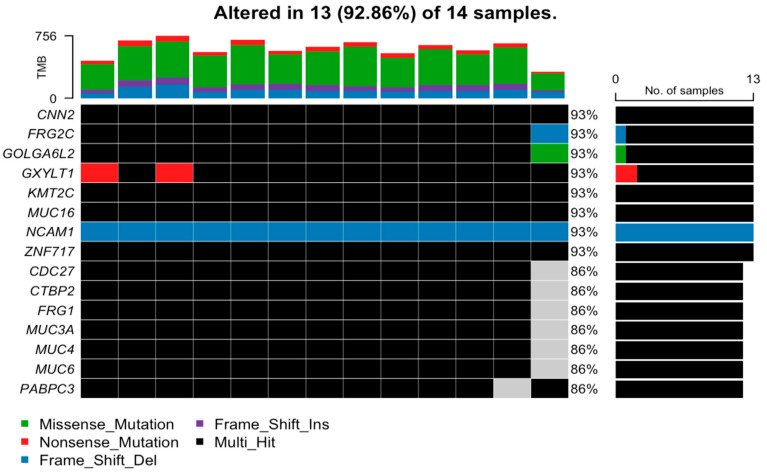
Oncoplot of our cohort showing the top mutated genes among the samples. Multi-hit mutations were observed in the majority of the patients. The *NCAM1* gene was deleted in 13 patients.

Copy number variant (CNV) analysis was not performed; hence, gene amplification analysis was outside of the scope of the study. An oncoplot displaying the most frequently mutated genes across the samples is shown in Figure 1. Missense mutations were the most frequent class of variants observed. Most of the variants were SNP. The highest proportion of the single nucleotide variants (SNV) were of the C>T class. The majority of the mutated genes are represented as multi-hit. These genes contain several alterations at the same time. *NOTCH*, *MYB*, and *SPEN* (*NOTCH* family) are not displayed in the oncoplot as a lower proportion of patients harbored these aberrations. The *NOTCH* pathway was aberrant in 35% of patients, of which one developed metastasis and one died of disease; 7.1% had mutations in *NOTCH1* (n = 1), 14.3% had *NOTCH2* (n = 2) or *NOTCH3* (n = 2) mutations. Mutations in *SPEN* were observed in 14.3% of samples (n = 2), namely a missense mutation and frameshift insertion. One of the patients with a *SPEN* mutation had metastasized and subsequently died of the disease. A single frameshift deletion was observed in the *NCAM1* gene, also known as CD56, in 13 patients. A nonsense mutation was displayed in the *GXYLT1* gene in two patients in the oncoplot and multi-hits for the other 11 patients. *KMT2C* was another highly multi-hit mutated gene harboring a single nonsense mutation in each sample and multiple missense mutations. 

**Figure 2:**
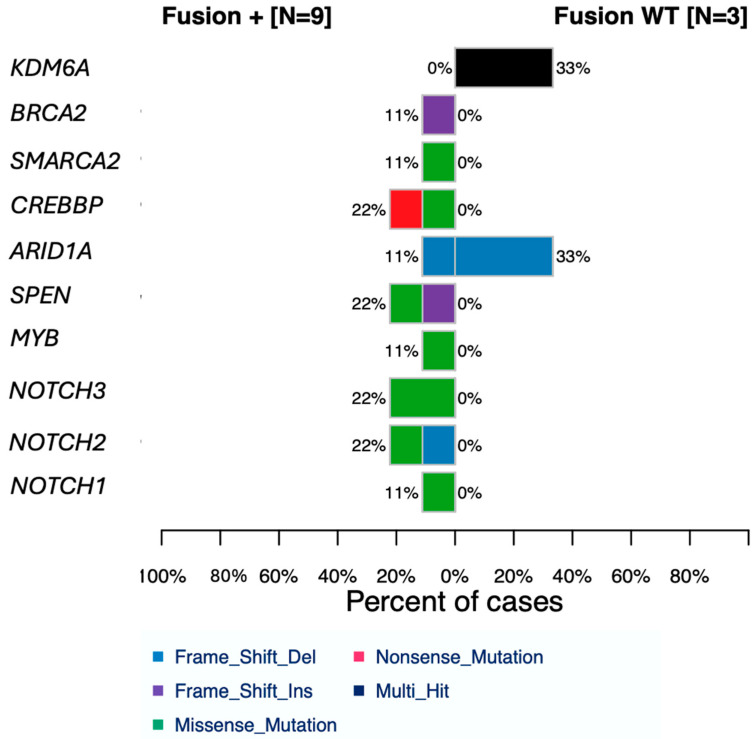
Cobarplot of a segregated cohort based on the presence (Fusion+) or absence (Fusion WT) of *MYB-NFIB* gene fusion. The DNA damage repair pathway was investigated to search for actionable aberrations allowing ACC tumorigenesis to occur.

Our patient samples were subsequently segregated by the presence or absence of *MYB-NFIB* gene fusion, as seen in Figure 2. ACC-associated genes, such as those affecting *NOTCH* signaling, are seen mutated in fusion-positive patients but not fusion-negative. Chromatin remodeling genes are affected. DNA damage repair (DDR) genes are mutated in fusion-positive patients; however, one wild-type patient also demonstrated a mutation in *ARID1A.* The significance of *MYB-NFIB* gene fusion on the genomic profile of ACC patients is highlighted. 

### 3.2. DDR Analysis

**Figure 3:**
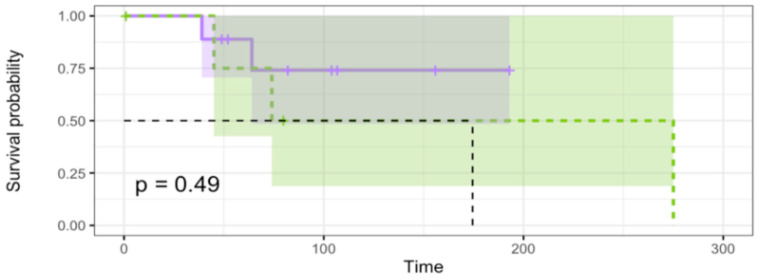
Survival analysis of the cohort based on mutations in the DDR pathway. Mutations included in the analysis were *ARID1A*, *CREBBP*, *BRCA1/2*, *ATM*, *POLD3*, *CHEK2*, *TP53*, and *PARP2*. No significant association with OS was obtained. Purple shaded line is DDR wild-type and green shaded line is DDR+.

The DNA damage repair pathway was interrogated in search of actionable aberrations allowing ACC tumorigenesis to occur. Literature searches were carried out seeking the most actionable DDR genes found mutated in cancers. Genes analyzed for the presence of mutations include *ATM*, *ATR*, *BRCA1/2*, *PALB2*, *RAD50*, *RAD51*, *PARP1/2/3*, *XRCC3*, *POLD1/2/3/4*, *POLE2/3/4*, *ARID1A*, *CREBBP*, *CHEK1/2*, and *TP53*. The frequency of DDR genes mutated in the cohort is represented in Figure 3. A small subset of the actionable mutations in genes of the pathway was observed in *ARID1A, CREBBP, BRCA2, CHEK2, PARP2*, and *POLD3*. No mutations were detected in *ATM, ATR, BRCA1*, and *TP53*, genes known to play an important role in the DDR. KM analysis was performed in relation to the DDR pathway, as shown in Table 4. Patients were segregated by the presence (DDR +) or absence (DDR WT) of mutations in the pathway. No association was determined between patients with an aberrant pathway and with a wild-type DDR pathway concerning survival. Hence, alterations in the pathway did not appear to have any bearing on survival. It is important to note that the total number of DDR mutated genes in our cohort occurred in nine patients. This means that some patients will have more than one DDR pathway mutation. 

Patient demographics for patients with DDR mutations can be found in Table 5. Two out of five patients harboring DDR mutations developed metastasis after a median of 51.5 months post-diagnosis, and three demonstrated *MYB-NFIB* gene fusion. They had a median age of 39 at diagnosis, and their OS was 77 months. Three patients with cribriform growth died. This is highlighted in the clinical profiles of the patients as they developed metastasis and died.

### 3.3. Survival Analysis

**Figure 4:**
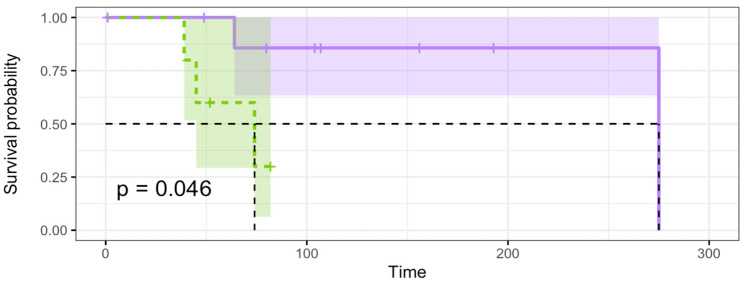
KM survival plot based on epigenetic status. Mutant epigenetic genes have a significant association with survival. Purple shaded line is wild-type and green dashed line is mutant epigenetic genes.

KM analyses were performed to identify an association between gender, age, metastasis, histology, and gene fusion with survival. KM plots were produced by stratifying clinical and genomic data to analyze survival based on defined parameters. The mean age for this analysis was 44 years. Patients with an age <45 were classified as young, whilst patients >45 were classified as old. There was no statistically significant difference in length of survival between the younger (<45) and older (>45) subgroups (*p* = 0.11). No statistical significance was found between gender and overall survival (OS) (*p* = 0.23). The presence of cribriform histology (*p* = 0.77), R/M occurrence (*p* = 0.73), and *MYB-NFIB* gene fusion status (*p* = 0.19) were not significantly associated with OS (*p* = 0.19). OS was significantly worse with the presence of mutations in epigenetic genes (*p* = 0.046) (Figure 4). The genes analyzed were *CREBBP*, *SMARCA2*, *KDM5A*, and *KDM6A.*

## 4. Discussion

This study aimed to uncover the clinical and genomic landscape of ACC, focusing on the *MYB*-NFIB fusion, *NOTCH* signaling pathway mutations, and epigenetic modifications.

FISH analyses confirmed the major contributory role of *MYB-NFIB* gene fusion in ACC tumorigenesis. The rearrangement of *MYB* with *NFIB* was seen in 76.9% of patients, which is in line with previous studies showing *MYB-NFIB* fusion in approximately 60% of ACC tumors [40]. Furthermore, both patients with a diagnosis of lacrimal gland ACC in our study possessed this gene fusion; 69.2% of patients had *NFIB* rearrangements, and 15.4% of tumor samples did not have rearrangements of *MYB* or *NFIB* genes on FISH analysis. This is in keeping with previous lacrimal gland ACC studies, whereby *MYB–NFIB* has been reported in between 50 and 80% of cases [41,42,43]. *MYB–NFIB* fusion has not been detected in other salivary carcinomas to our knowledge [40]. This specificity for ACC renders it an important potential biomarker and may be a very useful adjunct when attempting to establish a definitive diagnosis of a salivary gland neoplasm on histology [41].

Upregulation of *MYB* oncogene is known to occur in both fusion-positive and a subset of fusion-negative tumors [2]. This was observed in one patient who possessed *MYB* upregulation but lacked the *NFIB* rearrangement. *MYBL1* fusion with *NFIB* was not observed in our FISH analysis. *MYBL1-NFIB* fusion has been recently reported in lacrimal gland ACC for the first time, having been already observed in both breast and salivary gland ACC [14,43]. 

*MYB-NFIB* fusion status was not shown to be a statistically significant prognostic factor for overall survival, which correlates with previous studies [42]. Pharmacological research aimed at targeting *MYB–NFIB* fusion for ACC is lacking; however, a trial targeting MYB-NFIB fusion via Tet*MYB* vaccine and anti-programmed death 1 (PD1) antibody is currently in phase 1 [44]. 

The *NOTCH* signaling pathway is critical to development and homeostasis, and *NOTCH* pathway mutations promote tumorigenic processes such as cellular proliferation, tumor growth and survival, angiogenesis, and metastasis [45]. *NOTCH* pathway dysregulation has been observed in 11–29% of ACC tumor samples, and *NOTCH* signaling pathway mutations are associated with a more aggressive disease phenotype and an overall poor prognosis [30]. In our study, *NOTCH* pathway mutations were present in 41.6% of patient samples (Figure 2). 

*NOTCH* pathway mutations are associated with R/M, uncontrolled cellular proliferation, aggressive tumor subtypes, shorter survival time, and worse patient prognosis [20,22,25,30]. A recent study demonstrated that *NOTCH 1* mutations correlated with shorter relapse-free and overall survival compared to wild-type *NOTCH 1* [24]. This study highlights the need for the development of pharmacological therapies aimed at *NOTCH* signaling inhibition to ameliorate morbidity and mortality. 

It has also been demonstrated that *NOTCH* mutations can co-occur with other genetic mutations, including chromatin-modifying genes KDM6A, ARID1A, and CREBBP. This co-occurrence suggests that these mutations contribute to a broader pattern of genomic instability that drives disease pathogenesis [46]. 

Our study could not conclude that *NOTCH* played a role in patient overall survival and R/M status. Ho et al. recently conducted a comprehensive study of 1045 ACC tumor samples, of which 868 were recurrent. They demonstrated that *NOTCH* family and chromatin remodeling genes, including ARID1B and TERT promotor gene mutations, were overrepresented in recurrent/metastatic cases. Aberrations in *NOTCH*, *MYB*, and TERT promoters were able to identify four prognostic groups. They concluded that mutations in TERT promotor and *NOTCH* pathway genes are mutually exclusive. The groups were (1) Aberrant *MYB* and aberrant *NOTCH 1*, (2) Aberrant *MYB* and other, (3) Wild-type *MYB* and aberrant *NOTCH*, and (4) Wild-type *MYB* and aberrant TERT [30]. 

Group 1 was characterized by tumors that harbored both *MYB* fusions and *NOTCH1* mutations. Patients in this group had a more aggressive disease course and poorer prognosis. Patients in Group 2 possessed *MYB* fusions but lacked *NOTCH1* mutations. The prognosis for this group was variable and likely dependent on other genetic factors. Group 3 was characterized by tumors without *MYB* fusions but with *NOTCH1* mutations. Patients in this group had a poor prognosis due to the aggressive nature of *NOTCH1* mutations. Group 4 was characterized by a lack of both *MYB* fusions and *NOTCH1* mutations, but these patients possessed TERT promoter mutations. These mutations result in the activation of telomerase, thereby promoting tumor progression. The mutual exclusivity of TERT promoter mutations with both *NOTCH1* mutations and *MYB* fusions suggests that TERT activation represents an independent pathway of ACC oncogenesis. Patients in this group demonstrated a distinct clinical course when compared to the other three groups, with varying prognoses that depended on other concurrent genetic alterations [30]. The identification of these four prognostic groups underscores the complexity and heterogeneity of ACC. The mutual exclusivity of TERT promoter mutations with other genetic mutations indicates that TERT-targeted therapies may be effective for a subset of ACC patients. 

NOTCH1/2 aberrations have some overlap in regulating the effects of SPEN [47], a negative regulator of NOTCH signaling. SPEN mutations, similar to those in NOTCH, are associated with a poor prognosis in ACC patients [28]. In our study, 16.7% of patients demonstrated mutations in SPEN (Figure 2). SPEN mutations have also been associated with resistance to NOTCH pathway-targeted therapies, suggesting that SPEN mutations may contribute to therapeutic resistance and tumor progression [48].

The *GXYLT1* gene, another member of the *NOTCH* signaling pathway, was mutated in 93% of our patient samples (Figure 2). This xylotransferase gene is essential for proper *NOTCH* signaling [49]. Whilst studies on *GXYTL1* are limited, its role in the *NOTCH* pathway may be a driving force for subsequent *NOTCH1/2* aberrations, resulting in ACC tumor progression. 

The collective deregulation of the *NOTCH* pathway, through *NOTCH, SPEN*, or *GXYLT1* mutations, renders it a central mediator of ACC pathogenesis. Furthermore, the interplay between *NOTCH1*, *SPEN*, and *GXYLT1* highlights the need for personalized treatment strategies to be developed. Several *NOTCH* pathway inhibitors are undergoing clinical trials, including brontictuzumab, CB-103, and the selective gamma-secretase inhibitor AL101 [26,27,50].

The association of aberrations in the DDR pathway with ACC oncogenesis is novel in ACC studies (Table 4). Mutations in DDR genes allow for tumorigenesis to occur faster. DNA damage repair is vital for genomic stability; consequently, its loss of function leads to an increased risk of cancer. DDR is a group of mechanisms that sense DNA damage, signal its presence, and promote its repair in a substrate-dependent manner [51]. Without the presence of DDR, DNA lesions result in a blockade of metabolic processes such as transcription and replication or mutations, which would lead to senescence and cell death [51]. When DDR pathways malfunction and ACC driver mutations occur simultaneously, ACC proliferation occurs at a faster rate. The pathway is aberrant in a small subset of ACC patients, highlighting a niche mutational landscape. The dysregulated pathway as a result of the *BRCA2* mutation resulted in genomic instability, which allowed tumor progression and proliferation via the *NOTCH* pathway, as seen in the patient with a *BRCA2* mutation alongside an aberrant *NOTCH2* [52]. This is because the gene functions by mediating double-stranded break repair, genome stability, and transcriptional regulation [52]. It is postulated that aberrant *ARID1A* gene expression, which is normally involved in cellular processes, results in upregulated ACC cellular proliferation and impairment of the cell cycle DNA damage checkpoint [53]. DDR pathway mutations function to worsen genomic instability and thereby drive disease pathogenesis [4].

Epigenetic modifications function to upregulate tumorigenic pathways by controlling cellular pathways involved in tumor migration, tumor invasion, and growth [54]. Epigenetic modifications seen in neoplasia include chromatin modifications, histone acetylation, and non-coding RNA regulations [55]. The role of epigenetic modifications is poorly understood in terms of ACC pathogenesis. Our results identified chromatin remodeling alterations, including *SMARCA2*, *KDMA6*, and *CREBBP* (Figure 2). These proteins involve coding for subunits of chromatin remodeling complexes [53]. Chromatin remodeling mutations have previously been documented in salivary gland ACC tumor samples using whole-genome sequencing analyses and collectively may be present in up to 50% of ACC patients [28,29,53]. Previous studies in the literature report *CREBBP* mutations in 7% of ACC tumors, which is lower than our finding of 22% [53] (Figure 2). *CREBBP* aberrations were found in co-occurrence with *NOTCH*, DDR pathways, and other chromatin remodeling gene mutations. The mutations cluster within the helicase C domain, which, when mutated, increases susceptibility to neoplasia due to the disruption of the core DDR repair mechanisms. *CREBBP* is a known transcriptional co-activator of *MYB*, binding the central transactivating domain in *MYB* to modulate its function [30]. The concurrence of *CREBBP* with *NOTCH1*, along with the *MYB-NFIB* gene fusion, may be a key mutational driving force of ACC tumorigenesis [46]. *CREBBP* aberrations are also associated with increased *TP53* mutations. 

A notable chromatin remodeling gene, *CREBBP*, was found mutated in 16.7% of our patient cohort. A *NOTCH*1 mutation was observed alongside a nonsense mutation in *CREBBP*. This concurrence, along with the *MYB*-*NFIB* gene fusion, is hypothesized to be the driving force of ACC in this patient. *KDM6A* was mutated in 8.3% of patients in our study. These mutations are associated with poor prognosis. *KDM6A* and *KDM5A* are histone demethylases that play a critical role in the histone code. *KDM6A* specifically demethylates the H3 histone of Lys-27, whereas *KDM5A* demethylates the H3 histone of Lys-4. Both genes regulate DDR gene expression in cancers such as acute myeloid leukemia [56]. Mutations in *KDM6A* have been previously reported in lacrimal gland [43] and R/M ACC [29]. These mutations are associated with poor prognosis, highlighted by the development of metastasis in patients harboring a *KDM5A* alteration and the KM analysis. The occurrence of *NOTCH1* and *KDM6A* has been associated with poor prognosis; however, this co-occurrence was not observed in our study [30]. The *NCAM1* gene was found to be highly aberrant in the cohort. This gene encodes a protein involved in cell-to-cell interactions and cell–matrix interactions during differentiation and development. It regulates neurogenesis, neurite outgrowth, and cell migration whilst being involved in the expansion of immune cells necessary for immune surveillance, such as natural killer cells, highlighting the functional role of *NCAM1* in disease progression [57]. Further research is required to understand the role of *NCAM1* in ACC tumorigenesis. 

The mutational landscape of ACC suggests the requirement of mutated chromatin structures for transcriptional regulators to fully induce long-term changes to the cell’s phenotype. Mutations in *CREBBP*, *SMARCA2*, *KDM5A*, and *KDM6A* were associated with a significant reduction in overall survival in our patient cohort, supporting the idea that epigenetic and chromatin remodeling gene mutations confer a more aggressive disease phenotype and worse prognosis. 

Our results demonstrated no significant link between clinical and cytogenic profiles and survival in our cohort. The only parameter significantly associated with lower survival rates was epigenetic gene status (Figure 4), which is supported by previous studies in the literature [28]. A recent study by Brayer et al. highlighted the significance of histone modifications in ACC. They demonstrated that alterations in histone acetylation and methylation patterns were correlated with aggressive phenotypes and decreased overall survival [58]. 

### Study Limitations

This study has a number of limitations. Firstly, the study was limited to DNA sequencing. Copy number variant (CNV) analysis was not performed, and gene amplification analysis was outside of the scope of the study. CNV analysis may be informative in relation to diagnostics, predisposition to disease, and genetic marker identification by providing insight into the structural variations in the genome varying in copy number. 

Secondly, our small sample population limits any statistical power or significance garnered from this study. Further larger scale, multicentered studies are necessary to demonstrate relationships between certain gene abnormalities and clinical outcomes. 

## 5. Conclusions

In summary, ACC is a rare epithelial carcinoma with a generally low mutational burden that can arise in different anatomical locations with varying survival rates owing to differences in the genomic landscapes. Lacrimal gland ACC tumors are exceedingly rare but appear to have the same mutational spectrum as salivary gland ACC tumors. Due to the rarity of these tumors, it is difficult to be dogmatic about their epidemiology, and there is an unmet clinical need to characterize these tumors more efficiently and to develop targeted molecular therapies to treat this disease. In our study, the *NFIB* gene fusion was seen in 76.9% of patients and *NOTCH* pathway mutations in 35% of patients. DDR aberrations were found in a niche subset of the cohort. Epigenetic modifications significantly correlated with worse OS outcomes and worse prognosis. Further, large-scale studies are warranted to profile and characterize the molecular underpinnings of ACC further for the development of targeted therapies to reduce morbidity and mortality associated with the disease.

## Figures and Tables

**Table 1:** Filters applied to condense the MAF files.

Filters Applied	Threshold	Action
Read Depth	>30	Retained
Biotype	Protein coding	Retained
AF in general population	≤0.001	Retained
Variant classification	Frameshift, missense, nonsense	Retained
Consequence	Frameshift, missense, stop gain. Stop lost, start gain, start lost	Retained
Impact	Low	Removed
Filter	Common variants	Removed
PolyPhen	Benign	Removed
SIFT	Tolerated	Removed

**Table 2:** Patient demographics with median and percentage were calculated for 14 patients.

		Minimum	Maximum	Median
Age at Diagnosis (years)		20	72	42
Overall Survival (months)		39	275	85
Duration of Follow-up (months)		4	156	43.5
Time to Recurrence (months)		0	104	27
		N	%	
Overall Survival Status	Deceased	7	50	
	Alive/Censored	7	50	
Tumour Site	Salivary	12	85.7	
	Lacrimal	2	14.3	
Histological Pattern	Tubular	1	7.1	
	Cribriform	8	57.1	
	Solid	1	7.1	
	Mixed	4	28.6	
Recurrence/Metastasis		8	57.1	

**Table 3:** Patient demographics of 12 patients with FISH data. Abbreviations: Age (age at diagnosis in years), OS (overall survival in months), Follow-up in months, Time Rec (time to recurrence in months), OSS (overall survival status). In terms of histological grading, Grade 1 is Tubular, Grade 2 is Cribiform or mixed Tubular/Cribiform, and Grade 3 is any Solid component.

	Age	OS	Follow-Up	Time Rec	OSS	Tumor Site	Histology	Histology Grade
1	40	74	12	N/A	Deceased	Lacrimal	Solid (>30%), Cribiform and Tubular	3
2	33	275	125	84	Deceased	Salivary	Cribriform and Tubular, no Solid Component	2
3	42	193	156	0	Alive	Salivary	Mixed Solid (>50%), Cribriform and Tubular	3
4	44	156	153	N/A	Alive	Salivary	Mixed Cribiform and Tubular	2
5	66	39	1	0	Deceased	Salivary	Cribriform—no Solid	2
6	52	45	42	19	Deceased	Salivary	Cribriform—no Solid	2
7	72	64	6	35	Deceased	Salivary	Cribriform—no Solid	2
8	20	107	4	N/A	Alive	Salivary	Mixed—Cribriform and Tubular	2
9	32	90	90	N/A	Alive	Salivary	Cribriform—no Solid	2
10	33	80	1	N/A	Alive	Lacrimal	Tubular	1
11	34	42	42	37	Alive	Salivary	Cribriform—No Solid	2
12	53	31	31	1	Alive	Salivary	Mixed Solid (>30%)	3

**Table 4:** Frequency of DDR mutated genes in the cohort.

Mutated Genes	N	%
*ARID1A*	2	14.3
*CREBBP*	3	21.4
*CHEK2*	1	7.1
*BRCA2*	1	7.1
*POLD3*	1	7.1
*PARP2*	1	7.1

**Table 5:** Demographics of patients with mutations in the DDR pathway.

			Minimum	Maximum	Median
Age at Diagnosis (years)			33	52	39
Overall Survival (months)			45	275	77
Duration of Follow-up (months)			12	125	42
Time to Recurrence (months)			19	84	51.5
			N	%	
Overall Survival Status	Deceased		3	60	
	Alive/Censored		2	40	
Tumor Site	Salivary		3	60	
	Lacrimal		2	40	
Histological Pattern	Tubular		1	20	
	Cribriform		3	60	
	Solid		1	20	
Recurrence/Metastasis			2	40	
Gene Fusion		*MYB-NFIB*	3	60	
		*MYB*	1	20	

## Data Availability

The data can be shared up on request.
